# Specialism

**Published:** 1878-05

**Authors:** 


### EDITORIAL, ETC.

---

*Specialism.*—Drs. W. H. Atkinson, of New York, and J. E. Garretson, of Philadelphia, have lately issued a card, inviting correspondence from those interested in the formation of a Society of Oral Surgeons, "the object of which is the cultivation of the science and practice of oral surgery as a specialty in medicine." The card further states that "as the attainments necessary to fitness for the practice of such a specialty belong both to medicine and dentistry, the invitation is to those possessed of both qualifications." A meeting is proposed in New York City after responses to this call are received and considered.

We suppose this card to mean a Society of rather an exclusive sort, as it limits its membership to those holding medical as well as dental diplomas; though whether it is so worded in order to keep out the M. D's, or to exclude the simple D. D. S's, it does not appear. Why a man of attainments sufficient to practice dentistry as it should be practised, should be excluded from such an organization, we cannot really see; nor can we, considering the present ease with which medical diplomas are obtained all over the land, see why the addition of M. D. to his titles should give him consideration. If a "dental diploma is a sham," as the *Canada Dental Journal* says, the medical diploma is much more of a sham, and of itself is significant of nothing, save that the holder, perhaps, paid certain fees and possibly heard some lectures.

By all means form any Society which will give tone to the dental profession, and if you wish to do so, include in your practice as you certainly have the right, all operations in the oral cavity. But do not publish to the world a statement or an implication of a statement which is simply absurd: that of those now practicing dentistry, none are qualified to aid in organizing such a society as this card calls for, unless they can append the mystic M. D. to their names.

But there are hopes of the medical profession. Prof. Green, of Pennsylvania, in an address in New York, before the "American Academy of Medicine," represents an organization which has for its object the "bringing of those who are alumni of both classical (or scientific) and medical schools into closer relations with each other." The purpose for which this Society is formed is "to encourage young men to pursue regular courses of study in classical and scientific institutions before entering upon the study of medicine, and to extend the bounds of medical science and elevate the profession." The constitution further prescribes that fellows of the Academy shall be alumni of respectable institutions of learning, having taken the degrees of A. B. and A. M., in addition to or preliminary to the degree of M. D., and requires the candidate to study three years to get this last degree. Some unimportant exceptions are made to allow for equivalent preliminary training. How long it will take to lift the profession of medicine out of the slough into which it is fallen by such means it is hard to say, but we are glad that there are men willing to take steps which tend that way; it is full time. We hope to say more on this subject hereafter, and shall watch the "American Academy of Medicine" with curious interest.

